# Nevus with Intralymphatic Nevus Cell Protrusion and Lymphatic Invasion

**DOI:** 10.3390/diagnostics15182382

**Published:** 2025-09-18

**Authors:** Fanni Hegedűs, Zsuzsanna Ujfaludi, Orsolya Oláh-Németh, Tamás Lantos, Sándor Turkevi-Nagy, István Balázs Németh, Anita Sejben

**Affiliations:** 1 Department of Pathology, University of Szeged, 6725 Szeged, Hungary; 2 Competence Centre of the Life Sciences Cluster of the Centre of Excellence for Interdisciplinary Research, Development and Innovation, University of Szeged, 6720 Szeged, Hungary; 3 Department of Medical Physics and Informatics, University of Szeged, 6720 Szeged, Hungary; 4 Department of Dermatology and Allergology, University of Szeged, 6720 Szeged, Hungary

**Keywords:** nevus, intralymphatic nevus cell protrusion, lymphatic invasion

## Abstract

We hereby present a case of a 51-year-old woman with a pigmented nodule in the right axillary region. Histopathological examination revealed features consistent with an intradermal nevus. Notably, adjacent to the nevus, intralymphatic protrusion and lymphatic invasion were observed, comprising cells with morphological and immunohistochemical characteristics consistent with nevus cells. Next-generation sequencing revealed the *BRAF V600E* mutation. To date, 26 similar cases involving intralymphatic nevus cell protrusion and lymphatic invasion have been reported in the literature. Although this finding is rare and may pose a diagnostic challenge for pathologists, it should not be interpreted as indicative of malignancy. Rather, it must be assessed in the context of the lesion’s overall histological architecture.

**Figure 1:**
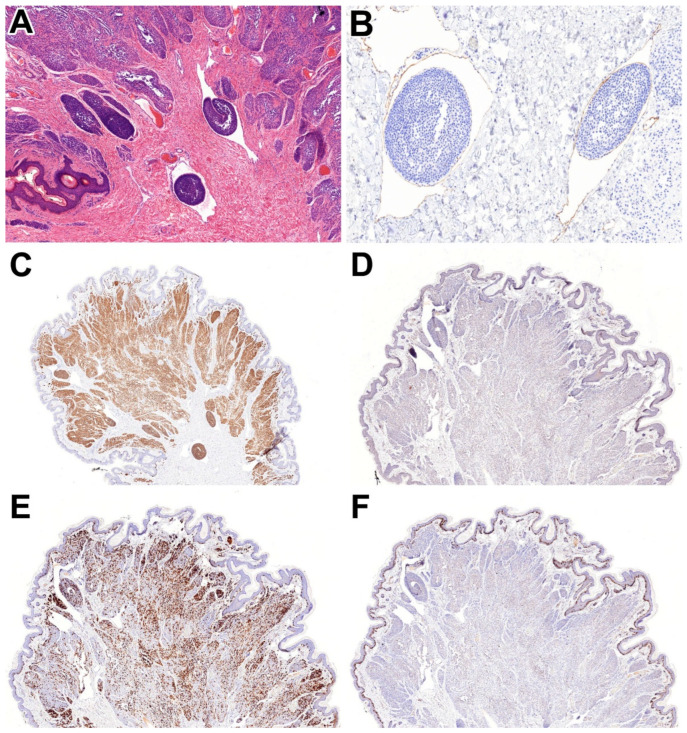
We present the case of a 51-year-old female patient with a well-circumscribed, homogeneous, brownish pigmented papule measuring 7 mm at its greatest dimension, located in the right axillary region, clinically suspected to be a pigmented naevus. The lesion was surgically excised, and histopathological examination revealed intradermal nests of nevus cells without evidence of cytological atypia, mitotic activity, or pagetoid spread. Adjacent to the nevus, protruding nevus cells or intraluminal nevus cell nests (**A**—HE, 5x) were identified in prominent lymphatic vessels (**B**—D2-40, 15x). CD31 and CD34 were negative. Immunohistochemically, the nevus cells exhibited diffuse cytoplasmic positivity for MelanA (**C**—2x), while PRAME staining was negative (**D**—2x). P16 demonstrated focal nuclear positivity (**E**—2x), and the Ki-67 proliferation index was below 1% (**F**—2x). A final diagnosis of intradermal nevus with intralymphatic nevus cell protrusion and lymphatic invasion was rendered. Next-generation sequencing revealed *BRAF V600E* mutation (variant allele frequency: 40.91%; tumour cell ratio: 60%). The lesion was completely excised; however, the patient was advised to attend a follow-up appointment in one year. At the time of this report, 3 months have elapsed since the surgery, and the patient has remained asymptomatic. Nevi are defined as benign clonal proliferations of nevus cells, according to the current World Health Organization (WHO) classification [1]. Intranodal nevus cells were first described in 1931 [2]. Since then, there have been several reported cases describing lymphatic protrusion of nevus cells, lymphatic invasion, and even nevus cell involvement within lymph nodes [3,4,5,6,7]. The presence of benign nevus cells in lymph nodes has been hypothesised to result from lymphatic dissemination, even in the absence of a detectable primary malignancy [8]. Although the current WHO classification considers lymphovascular invasion a hallmark of malignancy, such features have occasionally been observed in otherwise benign nevi [1]. The underlying mechanisms and clinical significance of these findings remain unclear. Currently, 8 articles are available detailing the above-mentioned phenomenon, encompassing 26 cases, summarised in Supplementary Table S1. A female predominance was observed among reported cases (*n* = 20; 74%), with a mean patient age of 23 years (median: 24; range: 1–51 years) [9,10,11,12,13,14,15,16]. The aetiology of these phenomena remains unclear. Leblebici et al. have proposed possible mechanisms, including mechanical transport of nevus cells and the theory of benign metastasis [15]. Subramony et al. described a case involving a breast cancer patient with subcapsular nevus cell aggregates in axillary lymph nodes, as well as nevus cells within the afferent lymphatics. Notably, an intradermal nevus excised during surgery also demonstrated histological evidence of lymphatic invasion [10]. Data on the duration of these lesions remains limited. Katsumata et al. reported a nevus persisting for several years, while Leblebici et al. documented three congenital nevi. For the remaining cases, the average lesion duration was 3.6 years (median: 3; range: 1–8 years) [12,15]. Lesion localisation was most commonly on the face (*n* = 8), followed by the back (*n* = 6), ear (*n* = 3), and cheek (*n* = 2). Single cases were identified on the conjunctiva, neck, breast, chest, gluteal region, thigh, and leg, respectively. The mean maximum macroscopic diameter of the lesions was 7 mm (median: 6; range: 2–16 mm), with one case described as “pea-sized” by Kim et al. [9,10,11,12,13,14,15,16]. Thickness was reported exclusively by Leblebici et al., with a mean of 3.4 mm (median: 2.9; range: 1.3–7 mm) [15]. Histologically, the nevi were classified most frequently as intradermal (*n* = 12), followed by compound (*n* = 9) and Spitz nevi (*n* = 7) [9,10,11,12,13,14,15,16]. Despite the majority of reports being dated, only three studies incorporated immunohistochemical analysis [12,15,16]. Katsumata et al. demonstrated S100 and vimentin positivity in nevus cells, whereas Leblebici et al. and Sood et al. confirmed the lymphatic endothelial lining using CD31 and D2-40, and CD34, respectively. Additionally, Leblebici et al. observed lymphangiectasia in 25% of their cases, which may represent a contributing factor in nevus cell lymphatic involvement. Molecular analyses have not been performed in the articles found in the literature. Some authors, however, mention DNA copy number change examination as a diagnostic tool to differentiate melanoma from melanocytic nevus [17,18,19]. Molecular examination has so far been carried out solely in our case. From a differential diagnostic standpoint, the most critical distinction to make is between this lesion and nevoid melanoma. Former articles showed an incidence of misdiagnosis of 22–50% [20,21]. An intraepidermal component—such as melanoma in situ—characterised by atypical junctional melanocytes arranged as single cells or in nests, with possible pagetoid spread or ulceration, may or may not be observed. Meanwhile, the intradermal component often appears ‘nevus-like’, typically showing a symmetric outline and well-defined lateral borders. Limited or absent dermal maturation—meaning a lack of the typical decrease in nest size and transition to single melanocytes with depth, as seen in benign nevi—is commonly observed. Features at low power that should raise suspicion for malignancy include increased cellularity with crowding, the presence of melanin pigment within deeper melanocytes, and/or elongated dermal nests arranged in parallel, often bordered by dense dermal collagen [22]. However, mitotically active nevi or nevi with so-called pseudomelanomatous features, such as marked fibrosis, are still considered difficult when it comes to differential diagnosis [23,24,25]. With immunohistochemical analysis, nevoid melanomas tend to show strong and diffuse positivity with PRAME, and a complete loss of p16 [1,26]. Genetic mutations so far have not been associated with naevoid melanoma [27]. The other main diagnostic pitfall is deep-penetrating/plexiform melanocytoma, a common mimic of malignant melanoma that may reflect histological features such as fascicular or plexiform proliferation of epithelioid melanocytes with vesicular nuclei and small nucleoli, typically accompanied by melanophages and representing the minor component of a combined nevus, and BRAF V600E positivity with immunohistochemistry, alongside PRAME negativity, and higher (>5%) Ki67 index, that may aid the final diagnosis. Furthermore, *BRAFV600E* or exon 3 *CTNNB1* mutations have been associated with deep-penetrating/plexiform melanocytoma [1,28,29,30,31]. Given the rarity of reports describing benign nevus cell invasion and dissemination, such findings should be interpreted with caution [8]. Intralymphatic nevus cell protrusion, as well as lymphatic invasion, is an extremely rare phenomenon that may be observed in nevi. Such cases should be handled with care, and potential differential diagnostic pitfalls, such as nevoid melanoma and deep-penetrating/plexiform melanocytoma, should be ruled out. This article reports the 27th documented case of this phenomenon in the literature and notably represents the first study to include molecular genetic analysis.

## Data Availability

The original contributions presented in this study are included in the article/Supplementary Materials. Further inquiries can be directed to the corresponding author.

## Supplementary Materials

The following supporting information can be downloaded at: https://www.mdpi.com/article/10.3390/diagnostics15182382/s1, Table S1: Results of the literature review.

## Abbreviations

The following abbreviations are used in this manuscript:

BRAF	v-Raf murine sarcoma viral oncogene homolog B
CD	Cluster of differentiation
CTNNB1	Catenin beta 1
NA	Not applicable
P16	p16INK4a protein
PRAME	Preferentially expressed antigen in melanoma
WHO	World Health Organization
