# Supplementary material for: Nevus with Intralymphatic Nevus Cell Protrusion and Lymphatic Invasion
Source: Diagnostics (Basel). 2025 Sep 18;15(18):2382. doi: 10.3390/diagnostics15182382 (PMC12468239; doi:10.3390/diagnostics15182382)
Supplement: Supplementary file 1 [file diagnostics-15-02382-s001.zip › diagnostics-3851593-supplementary.pdf]

First author	Year of publication	Number of patients	Gender of patient(s)	Age of patient(s)	Duration (years)	Localisation	Maximum diameter (mm)	Nevus thickness (mm)	Nevus subtype	Immunohistochemical analysis	Molecular examination	Other findings
Bell [9]	1979	5	Female (n=5), male (n=1)	5; 22; 29; 42; 45	NA	Back (n=2), conjunctiva (n=1), ear (n=1), skin, further not specified (n=1)	NA	NA	Compound	NA	NA	NA
Subramony [10]	1985	1	Female	41	NA	Breast	NA	NA	Intradermal	NA	NA	NA
Howat [11]	1985	7	Female (n=6), male (n=1)	1; 3; 6; 8; 8; 9; 13	NA	Cheek (n=2), ear (n=2), knee (n=2), thigh (n=1)	6 (n=1), 5 (n=3), 4 (n=1), 3 (n=1), 2 (n=1)	NA	Spitz	NA	NA	NA
Katsumata [12]	1990	1	Female	30	Several years, further not specified	Back	5	NA	Intradermal	S100 and vimentin positivity in nevus cells	NA	NA
Batistatou [13]	2014	1	Female	32	NA	Back	8	NA	Compound	NA	NA	NA
Kim [14]	2014	1	Male	26	NA	Back	"Pea-sized"	NA	Intradermal	NA	NA	NA

Leblebici [15]	2016	9	Female (n=5), male (n=5)	7; 8; 21; 24; 25; 26; 27; 30; 33; 39	Congenital (n=3), 1 (n=1), 2 (n=2), 3 (n=1), 4 (n=1), 5 (n=1), 8 (n=1)	Face (n=6), neck (n=1), gluteus (n=1), back (n=1), chest (n=1)	3 (n=2), 4, 6, 7, 8, 10, 11, 15, 16	1.3; 1.4; 2.5; 2.6; 2.7; 3; 3.5; 3.6; 6; 7	Intradermal (n=7), compound (n=3)	CD31, D2-40 for the identification of lymphatic vessels	NA	Lymphangiectasia (n=4)
Sood [16]	2018	1	Male	13	NA	Leg	10	NA	Intradermal	CD34 for the identification of lymphatic vessels	NA	NA
Our case	2025	1	Female	51	NA	Right axillary region	7	3.5	Intradermal	MelanA and scattered positivity with p16 in nevus cells, alongside Prame negativity, and <1% Ki-67. D2-40 for the identification of lymphatic vessels	*BRAF V600E*	NA

Table S1 - Results of the literature review

Abbreviations: NA - Not applicable
